# Gastrointestinal involvement by mantle cell lymphoma observed by endoscopy: A case report

**DOI:** 10.1097/MD.0000000000006321

**Published:** 2017-03-24

**Authors:** De-Ming Li, Yue-Ping Jiang

**Affiliations:** Department of Gastroenterology, The Affiliated Hospital of Qingdao University, Qingdao, Shandong, China.

**Keywords:** endoscopy, gastrointestinal, mantle cell lymphoma, ultrasonography

## Abstract

**Introduction:**

Mantle cell lymphoma (MCL) is a subtype of non-Hodgkin B-cell lymphoma, accounting for 6% of all non-Hodgkin lymphoma. The typical appearance of intestinal MCL is multiple lymphomatous polyposis, whereas presentation as protruding lesions is uncommon. We herein report the case of a 64-year-old male patient who was admitted to our hospital with epigastric pains. On endoscopy, submucosal neoplasma were identified in the gastric antrum, the duodenal bulb, and the rectum. On endoscopic ultrasonography (EUS) (OLYMPUS EUS EU-ME2, Miniprobe sonography), the lesions were homogeneously hypoechoic and originated from the submucous layer or muscularis mucosa. Pathological examination of biopsied specimens from the lesions of the rectum revealed diffuse lymphomatous proliferation and dense infiltration by monomorphic and small cleaved cells with irregularly shaped nuclei. On immunohistochemistry, the cells were positive for cyclin D1, CD20, CD21, SOX-11, and Bcl-2, but negative for CD3 and CD10; these findings were compatible with a diagnosis of MCL.

**Conclusion:**

The EUS characteristics initially led to the suspicion of digestive neuroendocrine tumors, since MCL presenting as submucosal tumors on EUS is rarely reported. We herein present this case to suggest clinician to include MCL in the differential diagnosis of submucosal intestinal lesions, as early diagnosis and timely treatment may improve patient prognosis.

## 1 Introduction

Mantle cell lymphoma (MCL) is a subtype of non-Hodgkin B-cell lymphoma, accounting for 6% of all non-Hodgkin lymphoma.^[1]^ The typical appearance of intestinal MCL is multiple lymphomatous polyposis, whereas presentation as protruding lesions is uncommon. We herein report a case of a 64-year-old male patient ultimately diagnosed with MCL who was admitted to our hospital with epigastric pains. We present this case to suggest clinician to include MCL in the differential diagnosis of submucosal intestinal lesions.

## 2 Methods

We collected this patient's medical records and reviewed the related literatures. Informed consent to participate in the study was obtained from the patient, and the protoco was approved by the Affiliated Hospital Ethics Committee of Qingdao University.

## 3 Clinical Summary

**Figure 1:**
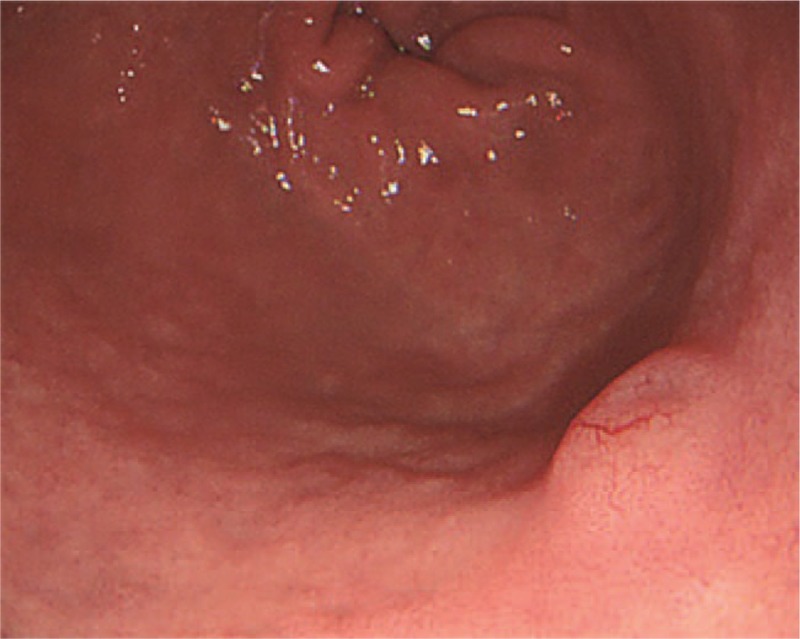
On endoscopy, several submucosal lesions were identified in the gastric antrum and duodenal bulb.

**Figure 2:**
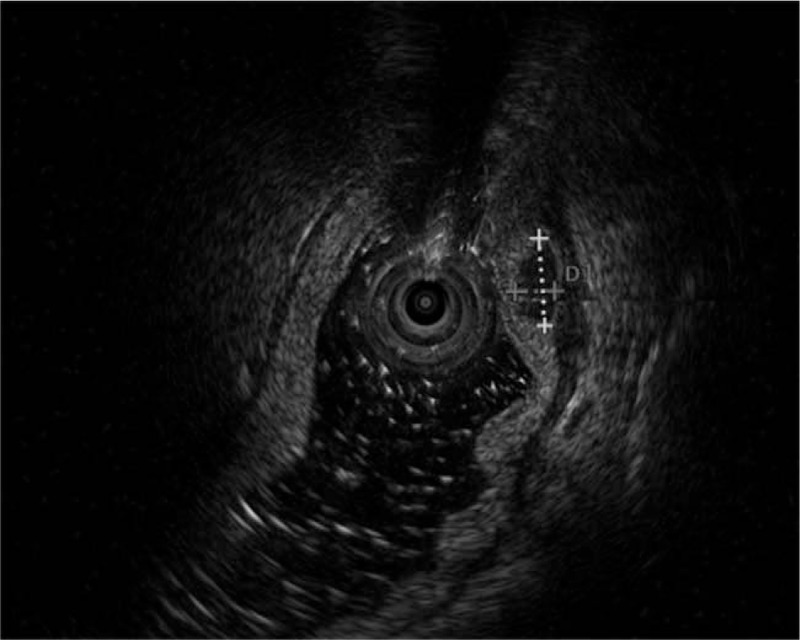
Endoscopic ultrasonography demonstrated that the lesions were almost 0.5-cm homogeneously hypoechoic neoplasms and originating from the submucous layer.

**Figure 3:**
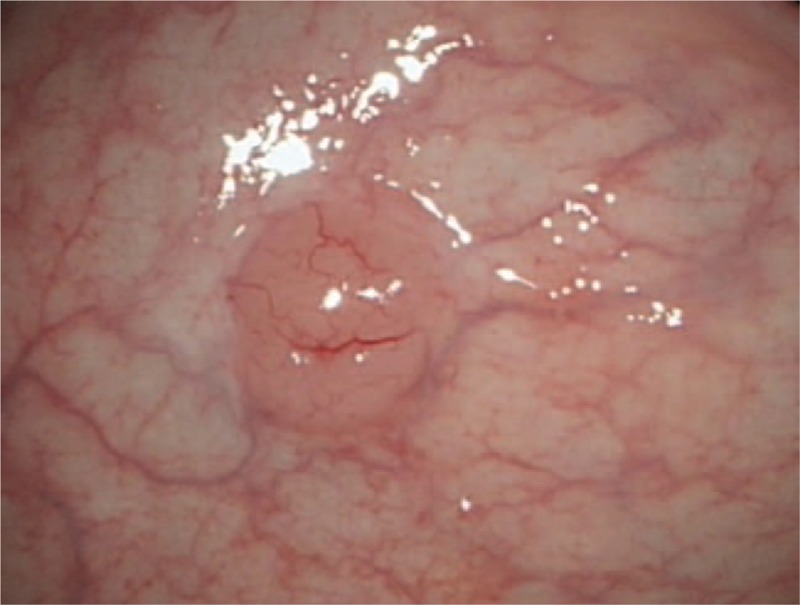
On endoscopy, multiple submucosal lesions in the rectum were identified.

**Figure 4:**
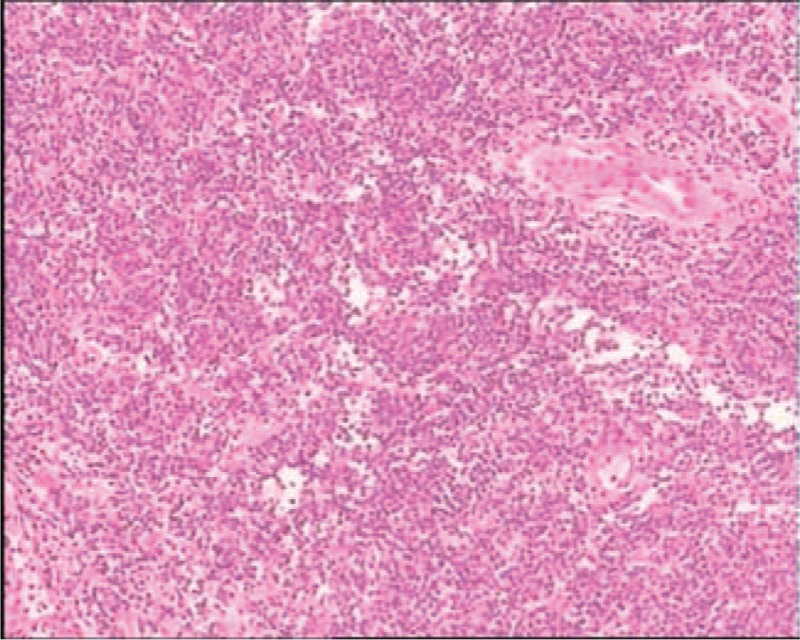
Pathological examination of the biopsied specimens from the lesions of the rectum revealed diffuse lymphomatous proliferation, with dense infiltration by monomorphic, small cleaved cells, with irregularly shaped nuclei.

A 64-year-old man was admitted to the Department of Gastroenterology of the Affiliated Hospital of Qingdao University Medical College (Qingdao, China) due to epigastric pains. Physical examination revealed no palpable mass, lymphadenopathy, or organomegaly. On endoscopy, several submucosal lesions were identified in the gastric antrum and the duodenal bulb (Fig. 1). Endoscopic ultrasonography (EUS) (OLYMPUS EUS EU-ME2, Miniprobe sonography) demonstrated that the lesions were almost 0.5-cm homogeneously hypoechoic neoplasms originating from the submucous layer (Fig. 2) and the initial diagnosis was digestive neuroendocrine tumors. Computed tomography revealed enlarged lymph nodes in multiple regions (mediastinal, retroperitoneal, mesenteric, and inguinal) and intracavitary nodules in the duodenum. To reach a definitive diagnosis, the patient underwent repeat EUS and biopsy was performed. On endoscopy, a 2 × 1-cm columnar uplift in the terminal ileum and multiple submucosal lesions in the rectum were identified (Fig. 3). EUS revealed that the lesions in the terminal ileum were sized 1.6 × 1.2 cm and the lesions in the rectum were sized almost 0.6 × 1.0 cm, they were all homogeneously hypoechoic and originated from the muscularis mucosa layer. Pathological examination of the biopsied specimens from the lesions of the rectum showed diffuse lymphomatous proliferation and dense infiltration by monomorphic, small cleaved cells with irregularly shaped nuclei (Fig. 4). On immunohistochemical analysis, the cells were positive for cyclin D1, CD20, CD21, SOX-11, and Bcl-2, but negative for CD3 and CD10, which was compatible with the diagnosis of MCL. Ki-67 staining revealed a proliferative index of 30%. Based on these findings, the diagnosis of Ann Arbor stage IV MCL was confirmed. The patient was referred for combination chemotherapy with rituximab, cyclophosphamide, doxorubicin, vincristine, and prednisone (R-CHOP regimen). The patient has been in remission clinically.

## 4 Discussion

MCL is a subtype of non-Hodgkin B-cell lymphoma, accounting for 6% of all non-Hodgkin lymphoma.^[1]^ The tumor cells are considered to originate from the mantle zone of the lymphoid follicle. The clinical symptoms of gastrointestinal involvement by MCL are nonspecific and may include vague abdominal pain, hematochezia, constipation, and diarrhea. The typical appearance of intestinal MCL is multiple lymphomatous polyposis, whereas presentation as protruding lesions is uncommon. In the present case, the lesions in the duodenum and rectum presented as submucosal neoplasms. On EUS, the lesions were homogeneously hypoechoic and originated from the submucosa while not affecting the propria. These characteristics may lead to misdiagnosis as digestive neuroendocrine tumors.

MCL is characterized by the chromosomal translocation t (11;14)(q13;q32), resulting in overexpression of cyclin D1, which plays a key role in cell cycle regulation and the progression of cells from the G1 to the S phase.^[2]^ To reach a definitive diagnosis, pathological and immunohistochemical analyses are required. As a minimally invasive method, EUS plays an important role in the diagnosis and management of the gastrointestinal submucosal tumors, as it clearly demonstrates tumor location, size, margin, echogenicity, and originating layer and also effectively identifies different tumors. However, although EUS has improved tumor visualization, more experience with the application of this modality must be accumulated. MCL mainly presenting as submucosal lesions on EUS has been rarely reported. We herein present this case to suggest clinicians to include MCL in the differential diagnosis of submucosal intestinal lesions, as MCL is associated with a poor prognosis^[3]^; early diagnosis and timely treatment may improve patient prognosis.
